# Draft genome sequence of *Castellaniella* sp. strain WN, a novel species capable of complete denitrification isolated from earthworm farm sludge

**DOI:** 10.1128/mra.00288-26

**Published:** 2026-07-10

**Authors:** Jingjing Wang, Hao Zhong, Mengdie Qi, Shujing Yang, Xiuying Li, Huijuan Jin, Jun Yan, Hongyan Wang, Yi Yang

**Affiliations:** 1 Institute of Applied Ecology, Chinese Academy of Sciences 74763, Shenyang, Liaoning, China; 2 University of Chinese Academy of Sciences 74519, Beijing, China; 3 Shenyang Agricultural University 98428, Shenyang, China; 4 Shenyang Tianfeng Biological Pharmaceutical Co., Ltd., Shenyang, China; 5 Key Laboratory of Forest Ecology and Silviculture, Institute of Applied Ecology, Chinese Academy of Sciences 74763, Shenyang, Liaoning, China; Wellesley College, Wellesley, Massachusetts, USA

**Keywords:** *Castellaniella*, denitrification, earthworm farm sludge, nitrogen cycle

## Abstract

The draft genome of denitrifying *Castellaniella* strain WN isolated from sludge of an earthworm farm comprises 3,730,778 bp with a guanine + cytosine content of 66.1%. The genome encodes 3,343 protein-coding sequences, 55 RNAs, and two copies each of the 5S, 16S, and 23S rRNA genes.

## ANNOUNCEMENT

The genus *Castellaniella* is generally associated with denitrification, a process that plays an important role in nitrogen cycling, although denitrification-associated genotypes may vary among strains (1). *Castellaniella* sp. strain WN was isolated in 2017 from a microcosm established with approximately 2 g of sludge collected from an earthworm farm in Shenyang, Liaoning Province, China (123.84°E, 42.72°N). Isolation was performed at 30°C by repeated streaking, spread plating, and liquid cultivation in bicarbonate-buffered (30 mM) basal mineral salt medium under anoxic conditions. Plate cultivation was performed in slant serum bottles with 2.5% agar in medium. Strain WN was transferred to fresh liquid medium containing 5 mM nitrate and able to reduce nitrate to nitrite and N_2_O; the subsequent depletion of N_2_O indicated its further reduction to N_2_.

Biomass was harvested from 1.6 L culture grown at 30°C to late exponential phase by centrifugation (15,000 × *g*, 15 min), and genomic DNA was extracted using HiPure Bacterial DNA Kit (Magen, China) without modification of the manufacturer’s protocol (2). The same DNA extract was used for sequencing on the PacBio Revio (HiFi) and Illumina NovaSeq Xplus PE150 platforms. For Illumina sequencing, a library with an average insert size of 350 bp was constructed using the NEBNext Ultra DNA Library Prep Kit (New England Biolabs, Ipswich, MA, USA) and sequenced on an Illumina NovaSeq Xplus PE150 instrument (Illumina, Inc., USA) with a paired-end approach (2 × 150 bp), generating a total of 20,380,136 paired-end reads (Q30 = 95.12%). Raw Illumina reads were quality-controlled and trimmed using fastp (3). For PacBio sequencing, the DNA sample was size selected with a 10-kb BluePippin (Sage Science, Beverly, MA, USA), and the library was prepared using the SMRTbell Template Prep Kit 3.0 (Pacific Biosciences, Menlo Park, CA). PacBio sequencing generated 64,909 long reads (*N_50_* = 14,530 bp). Default parameters were used for bioinformatics tools. Raw PacBio reads were processed using SMRT Link v10.1 for quality control, adapter trimming, and error correction (4). The quality of both PacBio long-read and Illumina short-read data sets was further assessed using FastQC (5). Raw reads were assembled using a hybrid approach with Unicycler v0.5.0, integrating PacBio and Illumina reads (6). Genome completeness and contamination were subsequently assessed using CheckM v1.1.3 (7).

The assembled genome of strain WN comprises three contigs, and its genomic features are summarized in Table 1. Gene annotation was performed using the National Center for Biotechnology Information Prokaryotic Genome Annotation Pipeline v6.9 (8). BLASTn analysis showed its 16S rRNA gene shared 99.3% similarity with *Castellaniella defragrans* strain DSM 12141 (NR_025280). Genome-to-Genome Distance Calculator analysis (9) revealed 41.1% digital DNA-DNA hybridization (dDDH) and 89.79% average nucleotide identity (ANI) between strain WN and *Castellaniella defragrans* strain DSM 12141 (GCA_014203015), indicating strain WN represents a distinct species (thresholds: 95% ANI and 70% dDDH) (10, 11). The genome encodes multiple denitrification-associated genes, consistent with the observed complete denitrification, and this pathway highlights the critical ecological role of strain WN in nitrogen cycling.

**TABLE 1:** Basic genomic features of *Castellaniella* sp. strain WN

Features	Value
No. of contigs	3
GC content (%)	66.1
Genome coverage	12×
Completeness (%)	98.29
Contamination (%)	0.03
Number of RNAs	55
Number of rRNAs (5S, 16S, and 23S rRNA)	Two copies
Number of identified genes	3,521
Number of identified CDSs	3,343
Nitrate reductase	*narI*, *narH*, and *narJ*
Copper-containing nitrite reductase	*nirK*
Nitric oxide reductase transcriptional regulator	*norR*
Nitrous oxide reductase	*nosL*, *nosD*, and *nosZ*

## Data Availability

Strain WN assembled genome has been deposited in GenBank under accession number GCA_054457555. The BioSample and BioProject numbers are SAMN50587050 and PRJNA1305117, respectively. The raw sequencing reads have been submitted to the Sequence Read Archive (SRA) under accession numbers SRR35020101 and SRR35023028 for PacBio and Illumina platforms, respectively.

## References

[1] Goff JL, Szink EG, Durrence KL, Lui LM, Nielsen TN, Kuehl JV, Hunt KA, Chandonia J-M, Huang J, Thorgersen MP, Poole FL 2nd, Stahl DA, Chakraborty R, Deutschbauer AM, Arkin AP, Adams MWW. 2024. Genomic and environmental controls on *Castellaniella* biogeography in an anthropogenically disturbed subsurface. Environ Microbiome 19:26. doi:10.1186/s40793-024-00570-9 38671539 PMC11046850

[2] Ashwini D, Tiwari SP. 2015. Use of CTAB method for isolation of good quality and quantity of DNA. J Pure Appl Microbio 9:2271–2274.

[3] Chen S, Zhou Y, Chen Y, Gu J. 2018. Fastp: an ultra-fast all-in-one FASTQ preprocessor. Bioinformatics 34:i884–i890. doi:10.1093/bioinformatics/bty560 30423086 PMC6129281

[4] Chin C-S, Alexander DH, Marks P, Klammer AA, Drake J, Heiner C, Clum A, Copeland A, Huddleston J, Eichler EE, Turner SW, Korlach J. 2013. Nonhybrid, finished microbial genome assemblies from long-read SMRT sequencing data. Nat Methods 10:563–569. doi:10.1038/nmeth.2474 23644548

[5] Andrews S. 2014. FastQC: a quality control tool for high throughput sequence data. Babraham Bioinformatics Institute, Cambridge, UK. https://www.bioinformatics.babraham.ac.uk/projects/fastqc/.

[6] Wick RR, Judd LM, Gorrie CL, Holt KE. 2017. Unicycler: resolving bacterial genome assemblies from short and long sequencing reads. PLoS Comput Biol 13:e1005595. doi:10.1371/journal.pcbi.1005595 28594827 PMC5481147

[7] Parks DH, Imelfort M, Skennerton CT, Hugenholtz P, Tyson GW. 2015. CheckM: assessing the quality of microbial genomes recovered from isolates, single cells, and metagenomes. Genome Res 25:1043–1055. doi:10.1101/gr.186072.114 25977477 PMC4484387

[8] Tatusova T, DiCuccio M, Badretdin A, Chetvernin V, Nawrocki EP, Zaslavsky L, Lomsadze A, Pruitt KD, Borodovsky M, Ostell J. 2016. NCBI prokaryotic genome annotation pipeline. Nucleic Acids Res 44:6614–6624. doi:10.1093/nar/gkw569 27342282 PMC5001611

[9] Meier-Kolthoff JP, Carbasse JS, Peinado-Olarte RL, Göker M. 2022. TYGS and LPSN: a database tandem for fast and reliable genome-based classification and nomenclature of prokaryotes. Nucleic Acids Res 50:D801–D807. doi:10.1093/nar/gkab902 34634793 PMC8728197

[10] Meier-Kolthoff JP, Auch AF, Klenk H-P, Göker M. 2013. Genome sequence-based species delimitation with confidence intervals and improved distance functions. BMC Bioinformatics 14:60. doi:10.1186/1471-2105-14-60 23432962 PMC3665452

[11] Jain C, Rodriguez-R LM, Phillippy AM, Konstantinidis KT, Aluru S. 2018. High throughput ANI analysis of 90K prokaryotic genomes reveals clear species boundaries. Nat Commun 9:5114. doi:10.1038/s41467-018-07641-9 30504855 PMC6269478

